# Breast Implant-Associated Anaplastic Large Cell Lymphoma: Case Report and Review of the Literature

**DOI:** 10.1155/2018/2414278

**Published:** 2018-01-21

**Authors:** Eva Berlin, Kunwar Singh, Christopher Mills, Ilan Shapira, Richard L. Bakst, Manjeet Chadha

**Affiliations:** ^1^Icahn School of Medicine at Mount Sinai, New York, NY 10029, USA; ^2^Department of Pathology, Mount Sinai Downtown, New York, NY 10003, USA; ^3^Department of Surgery, Mount Sinai Downtown, New York, NY 10003, USA; ^4^Department of Medicine, Mount Sinai Downtown, New York, NY 10003, USA; ^5^Department of Radiation Oncology, Icahn School of Medicine at Mount Sinai Hospital, New York, NY 10029, USA; ^6^Department of Radiation Oncology, Mount Sinai Downtown, New York, NY 10003, USA

## Abstract

We are reporting the case of a 58-year-old woman with history of bilateral silicone breast implants for cosmetic augmentation. At 2-year interval from receiving the breast implants, she presented with swelling of the right breast with associated chest wall mass, effusion around the implant, and axillary lymphadenopathy. Pathology confirmed breast implant-associated anaplastic large cell lymphoma (stage III, T4N2M0, using BIA-ALCL TNM staging and stage IIAE, using Ann-Arbor staging). The patient underwent bilateral capsulectomy and right partial mastectomy with excision of the right breast mass and received adjuvant CHOP chemotherapy and radiation to the right breast and regional nodes. Since completion of multimodality therapy, the patient has sustained remission on both clinical exam and PET/CT scan. We report this case and review of the literature on this rare form of lymphoma.

## 1. Introduction

Anaplastic large cell lymphoma (ALCL), a peripheral T cell lymphoma, represents approximately 2 to 3% of all non-Hodgkin's lymphomas [1]. Subtypes of ALCL can be grouped by the presence or absence of anaplastic lymphoma kinase (ALK) surface receptor and also by primary site of involvement, that is, primary cutaneous ALCL and breast implant-associated (BIA) ALCL [2]. BIA-ALCL is ALK negative, and similar to ALK positive and ALK negative ALCL, it is characterized by eccentric, horseshoe-shaped nuclei called “hallmark cells.” Unlike other types of ALCL, BIA-ALCL rarely invades beyond the breast. BIA-ALCL is distinct from primary breast lymphoma, which is composed of B cells and originates from the breast parenchyma.

The estimated incidence of BIA-ALCL is 2.03 per 1 million person years with an estimated prevalence of 1 per 30,000 women with breast implants [3]. The first case report of BIA-ALCL was published in 1997 by Keech and Creech [4]. In 2016, almost two decades later the World Health Organization labeled BIA-ALCL as a distinct entity [5]. Given widespread use of breast implants, there is an increased awareness of the risk for developing BIA-ALCL albeit rare.

In this paper, we describe a patient who was diagnosed with invasive BIA-ALCL two years after placement of silicone breast implants and was treated with multimodality therapy, that is, surgery, adjuvant chemotherapy, and locoregional radiation.

## 2. Case Report

**Figure 1:**
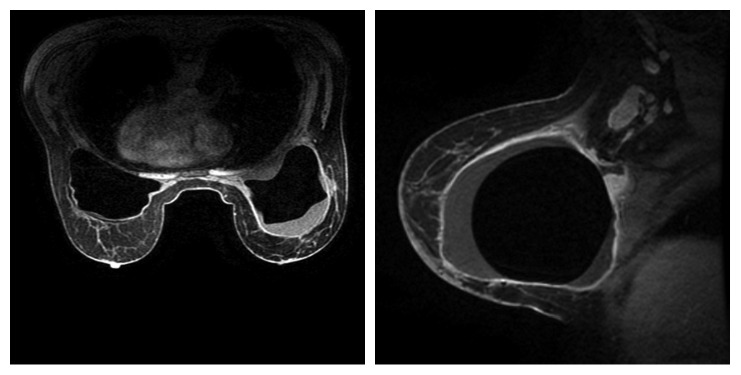
MRI at diagnosis showing fluid surrounding right breast implant, enlarged right axillary lymph node.

**Figure 2:**
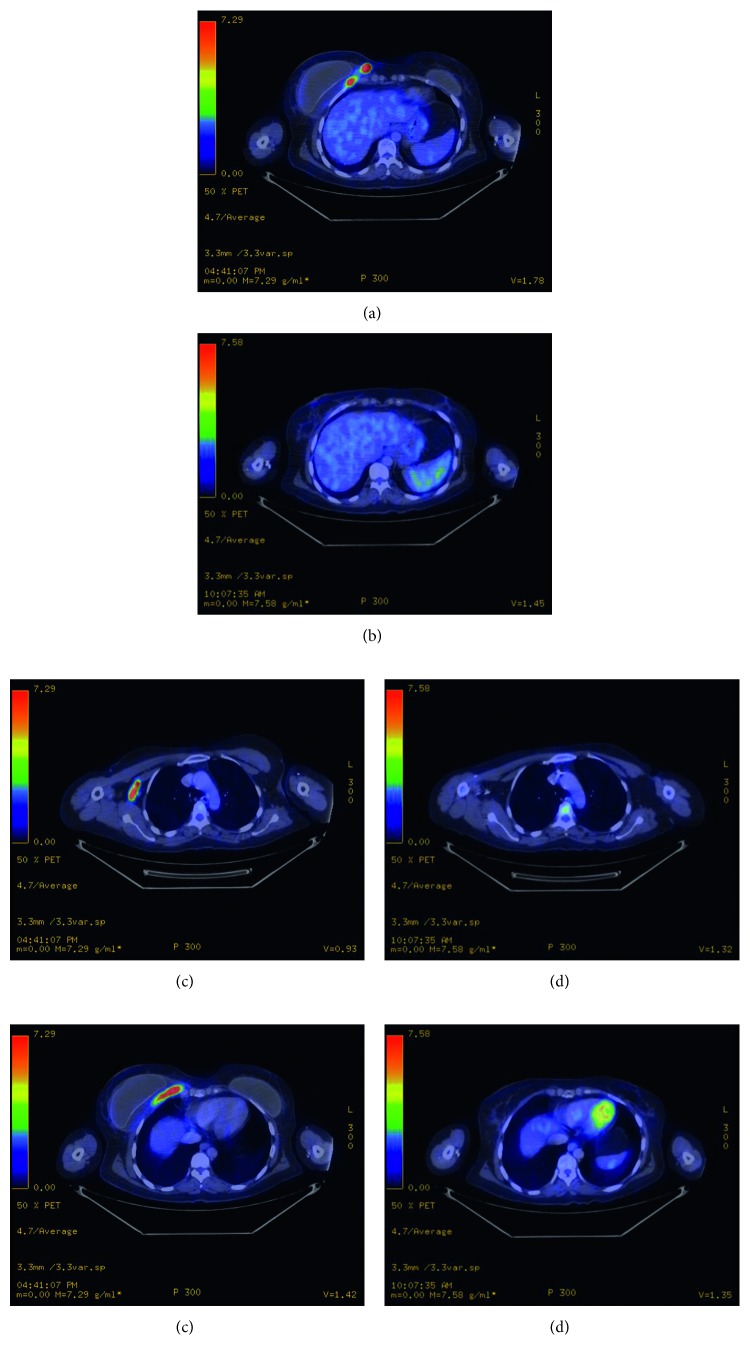
Pretreatment PET/CT staging scan showing right medial breast involvement (a) and restaging PET/CT after surgery and chemotherapy showing complete resolution of disease in the breast (b), pretreatment PET/CT showing right lymph node involvement (c) and posttreatment decrease in size of the nodes (d), and pretreatment right chest wall involvement (e) and posttreatment resolution of disease in chest wall (f).

**Figure 3:**
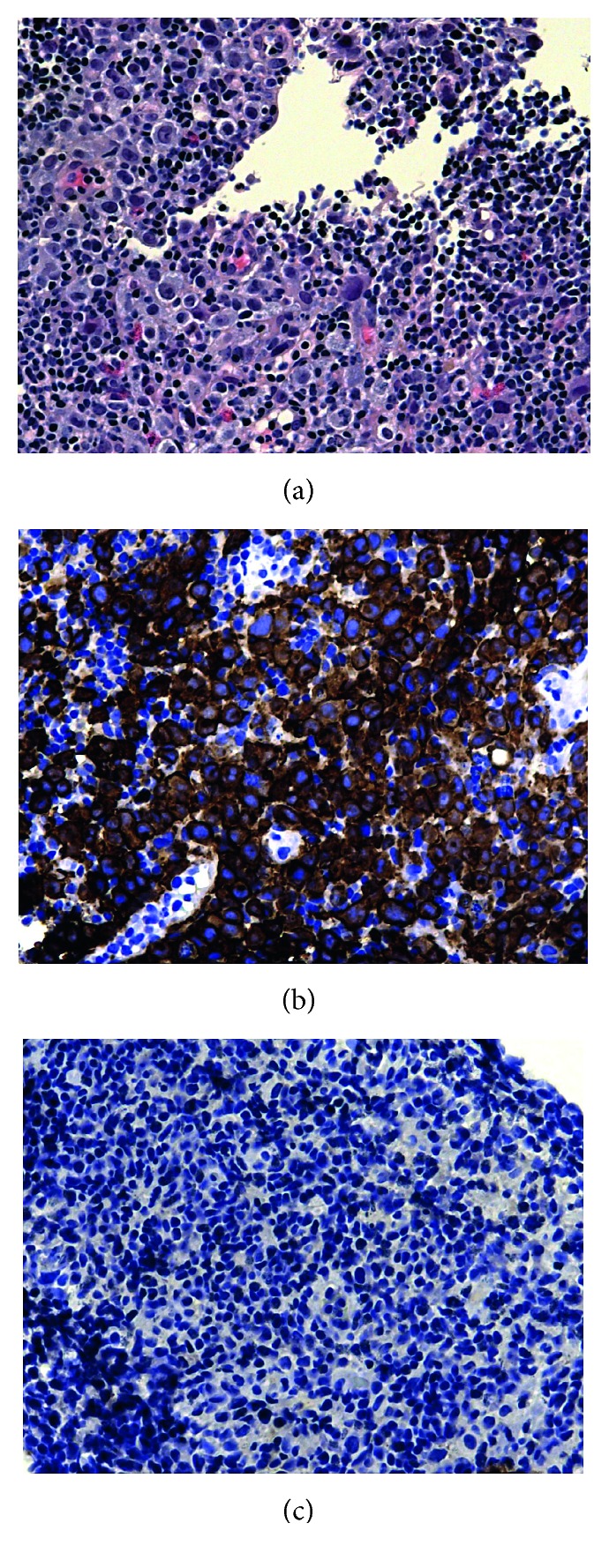
High power (40x) of horseshoe cells (cells with eccentric nuclei) with a mixed infiltrate in between, which are the “hallmarks cells” in anaplastic lymphoma (a), lymphoma/large cells shown diffusely positive for CD30 (b), and ALK shown diffusely negative throughout (c).

A 58-year-old female underwent bilateral breast lift and augmentation with silicone implants in September 2012. In August 2014, she presented with right breast swelling and heaviness in the inferior aspect of the breast. The patient denied weight loss, night sweats, fevers, chills, or systemic complaints. Imaging including mammogram and bilateral breast ultrasound noted presence of fluid surrounding the entire visible right breast implant. Presence of a 2 cm mass in the lower inner quadrant of the right breast and an enlarged 3 cm right axillary lymph node was also confirmed on MRI (Figure 1). Staging PET/CT showed multiple lesions in the right breast, the largest measuring up to 5 cm with SUV ranging from 10 to 52.4 (Figure 2) and two hypermetabolic lymph nodes in the right axilla measuring 1.7 and 3.7 cm with SUV of 6.0 and 15.8, respectively (Figure 2). There was also a hypermetabolic band posterior to the implant involving the pectoralis minor muscle measuring 5 × 1 cm with an SUV of 33.7 (Figure 2). Bone marrow biopsy was not performed. The biopsies of the breast, right axillary lymph nodes, and fluid cytology surrounding the breast capsule confirmed CD30+/ALK− anaplastic large cell lymphoma (Figure 3).

Multidisciplinary treatment plan included bilateral capsulectomy and right partial mastectomy with excision of the right breast mass and no axillary surgery. The right axillary node was intentionally left in place to serve as a correlate to the response to systemic chemotherapy during the postoperative period. At the time of surgery, all gross disease was removed; however, there was disease adherent to the chest wall in the area posterior to the dominant lesion of lower inner quadrant of the right breast. It was assumed that there would be residual microscopic disease in this area and that postoperative RT to the chest wall would be indicated, regardless of the results of a postoperative PET/CT scan. Surgical pathology noted residual lymphoma in the fibroconnective tissue of the right breast chest wall. In addition, surgical pathology confirmed areas of disease found on the pretreatment imaging (Figures 1, 2, 2, and e2(e)), including the right breast capsule, right breast extracapsular tissue, right medial breast glandular tissue, and right breast chest wall. Postoperatively, the patient received 6 cycles of adjuvant CHOP chemotherapy, which she tolerated well. PET/CT after 3 cycles of chemotherapy showed complete resolution of the previously noted hypermetabolic areas in the right breast (Figure 2) and chest wall (Figure 2), as well as a decrease in size of the right axillary lymph nodes to 0.5 cm (from 1.7 to 3.7 cm) (Figure 2). Subsequent to completing chemotherapy, the patient received radiation to the right breast and regional nodes. A total dose of 3600 cGy in 20 fractions using 180 cGy per fraction was delivered over 4 weeks. The patient tolerated the therapy well.

Since completing treatments, the patient has been followed by the multidisciplinary team at regular intervals and has not required subsequent therapy. At present follow-up 2.5 years from diagnosis, the patient remains without evidence of disease on physical exam and PET/CT scans.

## 3. Discussion

Advances in plastic surgery introduced the first breast augmentation procedure in 1962 [6]. Since then, there has been a steady rise in the number of patients undergoing breast augmentations. Worldwide, approximately 1.4 million breast augmentations were performed in 2015 [7]. In the United States, 290,467 breast augmentations were performed in 2016; this represented a 37% increase from 2000 [8].

BIA-ALCL most commonly occurs in patients of a median age of 52 years. The median time interval between breast implant and diagnosis is 9 years and ranges from 1 to 32 years [9]. Common presenting symptoms include swelling, pain, and redness of the affected breast [10]. Lymphadenopathy is a less common presenting symptom, found in one in 8 patients [11]. In most patients, BIA-ALCL occurs as a periprosthetic malignant effusion (∼70%), whereas the minority present with a capsular mass in addition to an effusion (∼30%) [9]. For staging and prognostication, the MD Anderson tumor, lymph node, metastasis (TNM) system of BIA-ALCL was proposed by Clemens et al. and is based on the American Joint Committee on Cancer TNM staging for solid tumors [12].

The pathogenesis of BIA-ALCL is unknown, but its association with textured implants is well recognized [3, 13]. By 2017, the FDA accumulated 359 medical device reports of BIA-ALCL with the majority of cases reported in textured implants versus smooth implants: 203 of the 231 medical device reports that included implant surface texture type were textured implants, and 28 were reported to be smooth implants [13]. Recent studies have theorized that chronic inflammation caused by bacterial biofilm of textured implants may mediate T cell hyperplasia and development of ALCL [14–16]. Hu et al. studied the biofilm response to implants in both pigs and humans and found that there was increased lymphocytic response in textured implants compared to smooth implants (*P* < 0.001), the lymphocytic infiltrate was primarily of T cell type, and there was a linear increase in the quantity of T cells and B cells in relation to the quantity of bacteria (*P* < 0.001) [15]. Loch-Wilkinson et al. also observed an association between increased surface area in textured implants and risk for developing BIA-ALCL. This suggests that environmental factors may trigger bacterial proliferation and T cell activation with chronic inflammation, resulting in increased risk of ALCL [16]. Kadin et al. proposed that BIA-ALCL is linked to chronic bacterial antigen stimulation of Th1/Th17 antigen-driven memory T cells in their study of biomarkers in the pathogenesis of BIA-ALCL [17].

In general, the prognosis of BIA-ALCL is excellent (Table 1). The patients have median survivals in the range 12 to 13 years. The FDA recorded 2.5% deaths from the 359 medical device reports [13]. Despite the overall excellent prognosis, there are certain presentations that are associated with worse prognosis. In a 2014 study by Miranda et al. of long-term follow-up of 60 patients with BIA-ALCL, those who had a mass were shown to have worse survival rates at 3 and 5 years compared to those without a mass [9]. A meta-analysis of 62 patients by Hart and Lechowicz noted that patients with a mass had an increased risk of death (*P*=0.043) and patients with extracapsular disease extension had an increased risk of recurrence (*P* < 0.001) and death (*P*=0.0008) [18]. In a 2016 study by Clemens et al., patients with lymphoma that spread beyond the capsule had a worse event-free survival [12].

Review of literature notes that among operable patients, total capsulectomy with removal of suspicious lymph nodes is the first line of treatment [12, 19, 20]. Among the 87 patients described in a study by Clemens et al., complete surgical excision (capsulectomy and implant removal) resulted in better overall survival and event-free survival compared to patients who underwent a limited surgery or treatment with systemic chemotherapy or radiation therapy [12]. A multidisciplinary approach is used in cases of BIA-ALCL that are not completely removed by surgery and patients presenting with high risk and advanced disease [20].  Table 2 is a summary of multidisciplinary treatment in patients presenting with mass and/or lymph node involvement and effusion. Postoperative radiation is given in high-risk patients including those with extensive disease, subtotal resection, positive margins, and chest wall invasion. In the case of locally advanced disease, lymph node involvement, or distant organ metastasis, systemic chemotherapy is given using either a combination of anthracycline-based chemotherapy (CHOP) or brentuximab vedotin (anti-CD30 antibody).

The management of patients with this rare disease has to be individualized. Our patient presented with high-risk features including effusion, chest wall mass, and lymphadenopathy received multimodality therapy and achieved an excellent response.

## Figures and Tables

**Table 1:** Summary of reported outcomes.

Reference	Total no. of patients	% Overall survival at 5 years	Median overall survival, years	% Treatments used
Miranda et al. [9]	60	92	12	93, complete surgical excision^∗^
				78, systemic chemotherapy^∗∗^
				55, radiation therapy^∗∗^
Clemens et al. [12]	87	89	13	49, limited surgery^∗∗∗^
				85, complete surgical excision
				58, systemic chemotherapy
				45, radiation therapy

^∗^Complete surgical excision includes total capsulectomy and implant removal. ^∗∗^Information for chemotherapy is available for 50 patients and for radiation therapy for 56 patients, percentages calculated from these respective totals. ^∗∗∗^Limited surgery is defined as partial capsulectomy, implant removal or replacement, or excisional biopsy of the capsule or mass.

**Table 2:** Summary of management of BIA-ALCL in patients presenting with mass and/or lymph node involvement and effusion alone.

Reference	Age, years	Effusion	Mass	Lymph node involvement	Treatments (given in order listed)
*Mass/node involvement*					
Zimmerman et al. [21]	48	Yes	No	Yes	6 cycles CHOP, bilateral total capsulectomy, 2 cycles salvage chemotherapy, 3 cycles anti-CD30 therapy with brentuximab vedotin, scheduled to receive stem cell transplant
Hwang et al. [22]	48	Yes	Yes	No	Unilateral capsulectomy, CHOP
Parthasarathy et al. [23]	43	No	Yes	Yes	2 cycles CHOP, cisplatin + gemcitabine, unilateral mastectomy with axillary nodes, RT (40 Gy in 15 fractions)
Tardio and Granados [24]	50	No	No	Yes	Bilateral capsulectomy, 4 cycles CHOP, RT
*Effusion only*					
Bautista-Quach et al. [25]	52	Yes	No	No	6 cycles CHOP
George et al. [26]	67	Yes	No	No	Bilateral capsulectomy
De Silva et al. [27]	38	Yes	No	No	Bilateral capsulectomy, RT (36 Gy in 20 fractions)
Smith and Ramsaroop [28]	33	Yes	No	No	6 cycles CHOP, bilateral capsulectomy
Wong et al. [29]	40	Yes	No	No	Bilateral capsulectomy, referred for CHOP and RT
Sorensen et al. [30]	59	Yes	No	No	Unilateral capsulectomy on affected side and bilateral removal of implants

CHOP, cyclophosphamide, hydroxydaunorubicin, vincristine, and prednisone/prednisolone; RT, radiation therapy; Gy, Gray.
